# The association of laminin levels with insulin resistance and non-alcoholic hepatosteatosis

**DOI:** 10.1186/s13098-021-00682-z

**Published:** 2021-06-06

**Authors:** Özgür Altun, Yücel Arman, Şengül Aydın Yoldemir, Ayşe Selcen Pala, Perihan Özkan Gümüşkaya, Mustafa Özcan, Mustafa Karataş, Okan Dikker, Tufan Tükek

**Affiliations:** 1 Department of Internal Medicine, University of Health Sciences, Prof. Dr. Cemil Taşçıoğlu City Hospital, Okmeydanı Eğitim ve Araştırma Hastanesi, Darülaceze Cad., No:27, PK: 34000 Şişli, Istanbul Turkey; 2 Department of Biochemistry, University of Health Sciences, Prof. Dr. Cemil Taşçıoğlu City Hospital, Istanbul, Turkey; 3 Department of Internal Medicine, Istanbul University, Istanbul Faculty of Medicine, Istanbul, Turkey

**Keywords:** Laminin, Insulin resistance, Non-alcoholic hepatosteatosis

## Abstract

**Background:**

Laminin, one of the largest glycoproteins of the basement membrane, is an important component of the extracellular matrix. Functions of the basement membrane include regulation of cell signaling behaviors and structural support. Laminin plays a critical role in the regulation of insulin action in muscle, liver, and adipose tissue. The study mainly investigates an association between the change in serum laminin levels and insulin resistance and non-alcoholic hepatosteatosis.

**Methods:**

This prospective study included a total of 90 participants; 60 patients diagnosed with Grade 2–3 non-alcoholic hepatosteatosis and 30 age- and sex-matched healthy controls between December 2019 and December 2020. Routine laboratory tests including glucose, insulin, homeostatic model of assessment-insulin resistance (HOMA-IR), alanine aminotransferase (ALT), aspartate aminotransferase (AST), triglyceride, low-density lipoprotein cholesterol (LDL-C), high-density lipoprotein cholesterol (HDL-C), and C-reactive protein and laminin levels were measured in the serum of the patient and control groups. Enzyme-linked immunosorbent assay was used for the measurement of laminin levels.

**Results:**

The median serum laminin levels were lower in patients with hepatic steatosis, compared to the control group (72 ng/L vs. 82 ng/L, respectively; p = 0.003). In the patients with insulin resistance, median laminin levels were lower, regardless of the presence of non-alcoholic hepatosteatosis (67 ng/L vs. 85 ng/L, respectively; p = 0.007). There was a weak, negative correlation between the laminin levels and HOMA-IR.

**Conclusions:**

Our study results suggest that, although there is no exact link between laminin and non-alcoholic hepatosteatosis, serum laminin levels are lower in patients with insulin resistance by regulating the insulin effect through integrins.

## Background

Insulin resistance occurs as a result of the decreased response to the effects of insulin in peripheral tissues (*i.e.* liver, adipose tissueand muscle) and is related to hyperinsulinemia. The ability of insulin to stimulate glycogen synthesis and suppress gluconeogenesis is diminished in the liver therebyincreasing hepatic glucose production [1]. Patients with insulin resistance usually have elevated asymptomatic liver enzymes and hypertriglyceridemia [1]. Non-alcoholic hepatosteatosisis defined as the accumulation of fat content, particularly triglycerides (TGs), greater than 5% of liver weight and is often associated with insulin resistance. In addition, macro- and micro-TG vesicles accumulate in hepatocytes without causing liver inflammation, liver cell death, or scarring [1, 2].

Laminin is an essential component of the extracellular matrix (ECM) and is one of the largest non-collagenous glycoproteins in the basement membrane. It consists of three polypeptide chains, *i.e.* alpha (α), beta (β), and gamma (γ) chains [3, 4]. It has a vital biological role including adhesion, displacement, cellular differentiation, growth, inflammatory response, and attachment to various components of the matrix [3, 4]. It was proposed that laminin is synthesized by hepatocytes and sinusoidal cells, whereas it is most commonly produced by stellate cells or lipocytes in sinusoids. Laminin receptors are located on the surface of a wide variety of cells, such as platelets, muscle cells, neutrophils, endothelial cells, and hepatocytes [3–5]. It is not found in hepatic parenchyma and sinusoids, since these tissues lack the basement membrane [6].

In addition, laminin plays a key role in fibrogenesis. Increased laminin levels are associated with hepatic fibrosis [7]. In various types of chronic liver disease (*i.e.* non-alcoholic fatty liver disease [NAFLD], alcoholic and viral liver diseases), it was shown to be linked to the severity of liver damage and degree of fibrosis [7–10]. However, it has not been routinely used as a fibrotic marker from early stages of fibrosis until the development of cirrhosis. In the literature, there area limited number of studies regarding laminin levels in the general population and in patients with non-cirrhotic NAFLD [10].

Given the fact that the ECM components and laminin emerge as key regulators of insulin action in muscle, liver, and adipose tissue [11] and exert their effects on the insulin mechanism through integrins, it is of utmost importance to examine the association of laminin, the main component of the ECM, with insulin resistance and non-alcoholic hepatosteatosis. In the present study, for the first time, we hypothesized that laminin might be associated with insulin resistance and non-alcoholic hepatosteatosis.Therefore, the aim was to investigate the association of serum laminin levels with insulin resistance and non-alcoholic hepatosteatosis.

## Methods

### Study design and study population

This prospective study was conducted at University of Health Sciences, Prof. Dr. Cemil Taşçıoğlu City Hospital internal medicine outpatient clinic between December 2019 and December 2020. Prior to the study, all participants were informed about the nature of the study and the option to withdraw from the study at any time for any reason. Written informed consent was obtained from each participant on a voluntary basis. The study was approved by the institutional Ethics Committee (No. 4867071-514.10) and conducted in accordance with the principles of the Declaration of Helsinki.

A total of 140 patients aged between 30 and 65 years who were admitted to our clinic for routine check-up were screened. Abdominal ultrasonography was performed in all patients. Grade 2–3 fatty liver was defined as non-alcoholic hepatosteatosis. Patients with non-alcoholichepatosteatosis (n = 60) were further divided into two equal groups including those without insulin resistance (Group B) and with insulin resistance (Group C). A Homeostatic Model of Assessment-Insulin Resistance (HOMA-IR) score of ≥ 2.7 was accepted as insulin resistance [12]. Patients with a history of any chronic disease, pregnant women, using steroids, with immobility, malignancy, chronic infection, diabetes, morbid obesity (body mass index [BMI] > 40 kg/m^2^), and chronic alcoholism were excluded from the study. Finally, a total of 90 participants, 30 without non-alcoholic hepatosteatosis or insulin resistance (Group A, control group), 30 with non-alcoholic hepatosteatosis and without insulin resistance (Group B), and 30 with non-alcoholic hepatosteatosis and insulin resistance (Group C) were included in the study.

Additionally, there was no fibrosis present in our patient group. We identified this using FIB-4 scoring. The fibrosis-4 (FIB-4) index for liver fibrosis score was calculated as age x AST (IU/I) / platelet count (10^9^/L) x √ ALT (IU/I) [13]. Every patient in the hepatosteatosis group was below the cut-off of 1.45 for the FIB-4 score used as fibrosis marker.

Demographic data including age, sex, weight, height, and BMI and laboratory test results including glucose, insulin, HOMA-IR, aspartate aminotransferase (AST), alanine aminotransferase (ALT), TG, low-density lipoprotein cholesterol (LDL-C), high-density lipoprotein cholesterol (HDL-C), and C-reactive protein (CRP) were recorded. HOMA-IR was calculated to evaluate insulin resistance using the following formula: serum insulin (uIU/mL) x serum glucose (mg/dL)/405 [12]. In addition to routine blood tests, additional blood samples were drawn from the volunteers into a biochemistry tube and kept for half an hour at room temperature, centrifuged at 4,000 rpm for 10 min, and the resultant serum was stored at − 80 °C until analysis.

### Measurements of laminin and performance characteristics of the laminin assay

The sera were thawed toroom temperature. An enzyme-linked immunosorbent assay (ELISA) kit (Catalog number: EA0058Hu) was used for the measurement of serum laminin levels. The analytical (linear) measurement range was set at 30 to 900 ng/L for laminin with a minimal detection limit of 15.38 ng/L. The reported intra-assay and inter-assay coefficient of variations (CVs) were 10% and 12%, respectively.

### Statistical analysis

The power analysis and sample size calculations were performed using the G*Power version 3.1.9.4 software (Heinrich-Heine-Universität Düsseldorf, Düsseldorf, Germany). The smallest sample size representing the universe at 95% study power was calculated as 21 with an effect size (d) of 1.05 and α margin of error of 5%, based on previous studies [10].

Statistical analysis was performed using SPSS version 20.0 (IBM Corp., Armonk, NY, USA). Descriptive data are expressed in mean ± standard deviation (SD) for continuous variables and in number and percentage (%) for categorical variables. The independent sample *t*-test was performed for the comparison of parametric data between two independent groups. The Mann–Whitney U test was used for the comparison of non-normally distributed variables. One-way analysis of variance (ANOVA) and Bonferroni *post-hoc* analysis were carried out for multiple comparisons of parametric variables, while the Kruskal–Wallis and Dunnett’s T3 *post-hoc* analysis were performed to test non-parametric variables. Univariate and multivariate regression analyses were performed to examine independent variables affecting the serum laminin levels. Logarithmic transformation was performed to ensure the normal distribution of laminin values and semi-logarithmic ordinary least squares models were used. A *p* value of < 0.05 was considered statistically significant.

## Results

Baseline demographic and laboratory data for the study groups are shown in Table 1. There was no statistically significant difference in the age and sex of the patient groups. The median serum laminin level was significantly lower in patients with non-alcoholic hepatosteatosis compared to those without non-alcoholic hepatosteatosis (72 [range, 64 to 86] ng/L *vs.* 82 [range, 78 to 109] ng/L, respectively; p = 0.003). In addition, the mean serum glucose, insulin, HOMA-IR, ALT, TG, and CRP levels were significantly higher and serum HDL-C levels were significantly lower in patients with non-alcoholic hepatosteatosis (p < 0.001 for both). However, there was no statistically significant difference in the mean AST and LDL-C levels between the groups (Table 1).

**Table 1:** Demographic and laboratory data of hepatosteatosis and control groups

	Control group (n = 30)	Non-alcoholic hepatosteatosis group (n = 60)	*P* value
Age (years), mean ± SD	43 ± 13.1	45.1 ± 9.8	0.455
Female, n (%)	22 (73.3)	41 (68.3)	NS
Male, n (%)	8 (26.7)	19 (31.7)	
BMI (kg/m^2^), mean ± SD	30.3 ± 4.8	32.01 ± 5.4	0.149
Glucose (mg/dL), mean ± SD	**86.8 ± 13.4**	**98.5 ± 11.5**	** < 0.001**
Insulin (mU/L), mean ± SD	**4.4 ± 3.2**	**12.9 ± 7.7**	** < 0.001**
HOMA-IR, mean ± SD	**0.95 ± 0.6**	**3.2 ± 2.1**	** < 0.001**
ALT (IU/L), mean ± SD	**15.7 ± 6**	**29.8 ± 18.2**	** < 0.001**
AST (IU/L), mean ± SD	19.2 ± 8.5	26.3 ± 20.1	0.068
TG (mg/dL), mean ± SD	**107.3 ± 30.2**	**197 ± 129**	** < 0.001**
LDL-C (mg/dL), mean ± SD	129 ± 53.6	129 ± 34.8	0.997
HDL-C (mg/dL), mean ± SD	**53.5 ± 9.5**	**45.5 ± 11**	**0.001**
CRP (mg/L), mean ± SD	**3.7 ± 4.8**	**4.5 ± 2**	**0.038**
Laminin (ng/L), median (25th–75th)	**82 (78–109)**	**72 (64–86)**	**0.003**^**a**^

^a^Mann-Whitney U, Others: Independent sample t-test. Data are given as mean ± SD, median or number and frequency, unless otherwise stated. P < 0.005 indicates statistical significance. *SD* standard deviation, *BMI* body mass index, *ALT* alanine aminotransferase, *AST* aspartate aminotransferase, *CRP* C-reactive protein, *HDL-C* high-density lipoprotein cholesterol, *HOMA-IR* Homeostatic Model of Assessment-Insulin Resistance, *LDL-C* low-density lipoprotein cholesterol, *TG* triglyceride, *NS* non-significant

The patients with non-alcoholic hepatosteatosis were further divided into two equal groups according to the presence of insulin resistance (Group B and Group C) and these groups were compared with the control group (Group A). The median serum laminin level was significantly lower in patients with non-alcoholic hepatosteatosis with insulin resistance (Group C) compared to those without insulin resistance (Group B) (67 [range, 63 to 74] ng/L *vs.* 85 [range, 67 to 109] ng/L, respectively; p = 0.007). However, there was no significant difference in the median serum laminin levels between the patients without non-alcoholic hepatosteatosis and insulin resistance (Group A) and patients with non-alcoholic hepatosteatosis and without insulin resistance (Group B) (82 [range, 78 to 109] ng/L *vs.* 85 [range, 67 to 108] ng/L, respectively; p = 0.772) (Table 2).

**Table 2:** Comparison of hepatosteatosis groups with and without insulin resistance and the control group

	Group A (control group)Hepatosteatosis(−) and HOMA-IR < 2.7) (n = 30)	Group B Hepatosteatosis ( +) and HOMA-IR < 2.7) (n = 30)	Group C Hepatosteatosis ( +) and HOMA-IR ≥ 2.7) (n = 30)	A–B–C	A–B	A–C	B–C
Laminin (ng/L) median (25th–75th)	**82 (78–109)**	**85 (67–109)**	**67 (63–74)**	**0.002**^**a**^	0.772^b^	**0.004**^**b**^	**0.007**^**b**^
Age (years), mean ± SD	43 ± 13.1	44 ± 9.4	45.3 ± 10.3	0.701	1.000	1.000	1.000
BMI (kg/m^2^), mean ± SD	30.3 ± 4.8	32 ± 4.5	32.8 ± 6.2	0.183	1.000	0.207	0.753
Glucose (mg/dL), mean ± SD	**86.8 ± 13.4**	**93.0 ± 8.2**	**104 ± 11.7**	** < 0.001**	0.114	** < 0.001**	**0.001**
Insulin (mU/L), mean ± SD	**4.4 ± 3.2**	**7.4 ± 2.2**	**18 ± 7.4**	** < 0.001**	0.059	** < 0.001**	** < 0.001**
ALT(IU/L), mean ± SD	**15.7 ± 6**	**28 ± 16.7**	**31.4 ± 19.7**	** < 0.001**	**0.007**	** < 0.001**	1.000
AST(IU/L), mean ± SD	19.2 ± 8.5	27 ± 26	24.6 ± 10.2	0.144	0.156	0.675	1.000
TG(mg/dL), mean ± SD	**107 ± 30**	**178 ± 116**	**208.3 ± 141.3**	**0.002**	0.041	**0.002**	0.835
LDL-C (mg/dL), mean ± SD	129 ± 53	136 ± 32	122.0 ± 35.9	0.426	1.000	1.000	0.578
HDL-C (mg/dL), mean ± SD	53.5 ± 9.5	**46 ± 10**	**44.2 ± 11.09**	**0.003**	0.048	**0.003**	1.000
CRP (mg/L), mean ± SD	3.7 ± 4.8	4.05 ± 1.6	3.7 ± 4.8	0.503	1.000	0.730	1.000

A: Non-hepatosteatosis and non-insulin-resistant control group; B: Hepatosteatosis, non-insulin-resistant group; C: Patient group with hepatosteatosis and insulin resistance. P < 0.005 indicates statistical significance. ^a^Kruskal-Wallis test (p < 0.05 significant); ^b^Dunnett T3 post-hoc analysis (significant at p < 0.017). One-way ANOVA (significant at p < 0.05) for multiple comparisonsand Bonferroni post-hoc analysis for binary comparisons (significant at p < 0.017). Data are given asmean ± SD, median or number and frequency, unless otherwise stated. *SD* standard deviation, *BMI* body mass index, *ALT* alanine aminotransferase, *AST* aspartate aminotransferase, *CRP* C-reactive protein, *HDL-C* high-density lipoprotein cholesterol, *HOMA-IR* Homeostatic Model of Assessment-Insulin Resistance, *LDL-C* low-density lipoprotein cholesterol, *TG* triglyceride

The multivariate regression analysis revealed an inverse relationship between serum laminin, HOMA-IR, and LDL-C levels (*r* = − 0.025 p = 0.006 and *r* = − 0.001 p = 0.007, respectively). However, there was no significant correlation between serum laminin levels and non-alcoholic hepatosteatosis (*r* = − 0.038 p = 0.321) (Table 3).

**Table 3:** Correlation analysis results

Variable	Serum laminin levels^a^							
	Univariate regression analysis			Multivariate regression analysis				
	*r*	*P* value	ß	SE	ß	95% CI		*P* value
Age	− 0.154	0.147						
BMI	− 0.003	0.978						
Sex	0.044	0.680						
Hepatosteatosis	− **0.287**	0.006^*^	− **0.038**	0.038	− 0.113	− 0.114	0.038	0.321
HOMA-IR (µIU/mL)	− **0.351**	0.001^*^	− **0.025**	**0.003**	− **0.325**	− **0.042**	− **0.008**	0.006^*^
ALT (IU/L)	− 0.183	0.084						
AST	− 0.165	0.123						
LDL-C	− **2.226**	0.034^*^	− **0.001**	** < 0.001**	− **0.270**	− **0.002**	** < 0.001**	0.007^*^
HDL-C	0.101	0.347						
TG	− 0.165	0.115						
CRP	− 0.105	0.349						

^a^Logarithmic transformation was performed to ensure the normal distribution of serum laminin level. *p < 0.05 indicates statistical significance. *SD* standard deviation, *SE* standard error, *CI* confidence interval, *BMI* body mass index, *ALT* alanine aminotransferase, *AST* aspartate aminotransferase, *CRP* C-reactive protein, *HDL-C* high-density lipoprotein cholesterol, *HOMA-IR* Homeostatic Model of Assessment-Insulin Resistance, *LDL-C* low-density lipoprotein cholesterol, *TG* Triglyceride

**Fig. 1:**
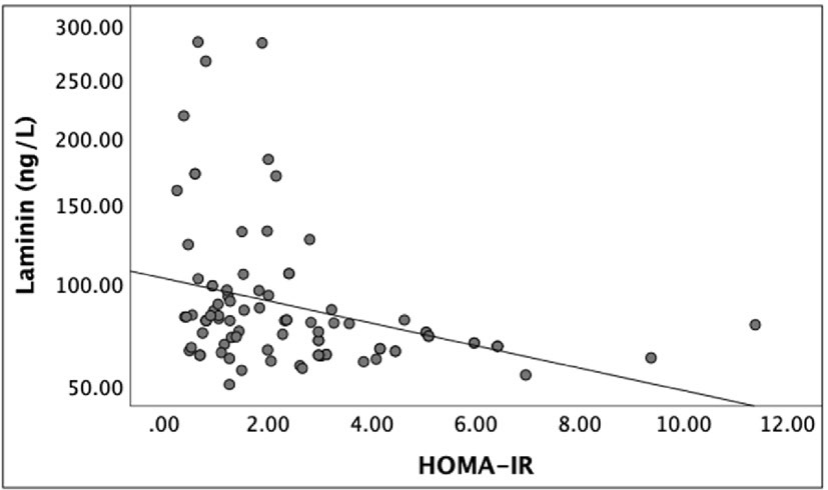
The scatter plot of changes between serum laminin levels and HOMA-IR. *HOMA-IR* Homeostatic Model of Assessment-Insulin Resistance

Figure 1 shows the scatter plot of changes between serum laminin levels and HOMA-IR. Accordingly, there was a weak, negative correlation between laminin and HOMA-IR levels, indicating that lower laminin levels were associated with higher HOMA-IR levels.

## Discussion

Non-alcoholic fatty liver disease is the leading cause of chronic liver disease in western countries [1]. It may present in a wide histological spectrum, ranging from simple steatosis to non-alcoholic steatohepatitis and can even progress to cirrhosis [1]. Systemic and hepatic insulin resistance play a central role in its etiology, and NAFLD is considered the hepatic sign of metabolic syndrome. Patients with NAFLD typically present with dyslipidemia, particularly hypertriglyceridemia and low HDL-C levels [2]. In addition, elevated liver enzymes were shown to be associated with increased CRP values [1, 2]. In the current study, we examined the role of serum laminin levels in the pathogenesis of insulin resistance and non-alcoholic hepatosteatosis. As expected, our results showed that the mean serum TG, ALT, CRP, and HOMA-IR levels were significantly higher in the patients with non-alcoholic hepatosteatosis compared to the control group, while the mean HDL-C levels were significantly lower in this patient group. Furthermore, we found significantly lower median serum laminin levels in patients with non-alcoholic hepatosteatosis compared to those without. However, the comparison of the patients with non-alcoholic hepatosteatosis without insulin resistance and the control group without non-alcoholic hepatosteatosis revealed no significant difference in the median laminin levels. The discrepancy in serum laminin levels can be attributed to insulin resistance, rather than the presence of non-alcoholic hepatosteatosis.

Previous studies demonstrated that laminin is a fibrosis biomarker in alcoholic liver disease, viral hepatitis, and NAFLD. In particular, high laminin levels were shown to be associated with the severity of fibrosis and hepatitis [7–10]. In a study investigating the clinical significance of the immunoreactive triple helical domain of type IV collagen in serum, Hirayama et al. [7] found a positive correlation between serum laminin levels and the development of liver fibrosis. Some other authors also suggested that serum laminin levels can be a reliable biomarker in the evaluation of liver fibrosis stages [8, 9]. In the literature, however, there is only one study including NAFLD patients. Santoz et al. [10] reported that serum laminin levels were significantly higher in NAFLD patients who developed fibrosis, compared to those without fibrosis.

In the present study, we found no significant relationship between the laminin levels and non-alcoholic hepatosteatosis. Therefore, we cannot speculate that laminin is a biomarker fornon-alcoholic hepatosteatosis. This can be explained in two ways: liver parenchyma lacks laminin; however, in advanced stages of liver disease, laminin was shown to be progressively deposited following pathological processes such as capillarization of hepatic sinusoids and fibrosis [10, 14]. Therefore, serum laminin levels may not increase at the time of hepatosteatosis, when fibrosis has not yet occurred. Also, the increase in serum laminin levels in liver cirrhosis was attributed to reduced destruction in addition to capillarization in the Disse’s space [14]. In light of these findings, serum laminin levels may not be elevated during the stages of non-alcoholic hepatosteatosis, since liver fibrosis has not yet occurred.

The interaction between the ECM and cells is of vital importance for all organs of the human body. ECM remodeling is associated with insulin resistance in several metabolic tissues. However, few studies addressed the link between ECM and glucose metabolism in the literature. Currently, it is still unclear how laminin contributes to alterations in insulin metabolism. One of the most widely adopted theories is that laminin regulates the effect of insulin through structures involved in integrin and integrin signalization [11]. Integrins are a family of adhesion receptors that mediate matrix and cell linkage [15]. They were shown to increase the effect of insulin by phosphorylation of focal adhesion kinase (FAK), a tyrosine kinase [11]. Increased FAK levels were found to increase glucose uptake and insulin sensitivity through insulin receptor Glut-4 translocation and FAK deficiency was associated with increased insulin resistance and TG levels [16, 17].

A review of the literature reveals no head-to-head clinical study showing the exact relationship between laminin and effects of insulin. However, in an experimental study, Hammar et al. [18] reported that laminin increased the FAK levels in their experimental study. Through this mechanism, laminin may provide beneficial effects by increasing insulin sensitivity. In our study, among the patients with non-alcoholichepatosteatosis, serum laminin levels were significantly lower in those with insulin resistance compared to those without insulin resistance. We also found a negative correlation between serum laminin levels and HOMA-IR. Consistent with those of Hammar et al. [18], these findings clinically indicate that low laminin levels may reduce the beneficial effects of laminin on the insulin metabolism. We, therefore, believe that low levels of laminin play a critical role in the development of insulin resistance. However, further experimental and clinical studies are needed regarding integrins and FAK which mediate the effects of laminin in insulin resistance.

There are some limitations to this study. The diagnosis of non-alcoholic hepatosteatosis was made based on the ultrasonography, rather than liver biopsy. In addition, the sample size in the group with insulin resistance is limited to draw firm conclusions.

The main strength of this study is that it is the first clinical study to investigate the relationship between serum laminin levels and insulin resistance. We believe that these findings will provide additional information forthe limited body of knowledge on this topic in the literature and an insight into the effects of laminin on the mechanism of insulin resistance.

## Conclusions

In conclusion, our study results suggest that although there is no exact link between laminin and non-alcoholic hepatosteatosis serum laminin levels are lower in patients with insulin resistance possibly by regulating the insulin effect through integrins. Targeting laminin may be beneficial for pathogenesis and associated complications of insulin resistance. However, further large scale, prospective studies are still warranted to draw a firm conclusion.

## Data Availability

The datasets used and/or analyzed during the current study are available from the corresponding author upon request.

## Abbreviations

ALT: Alanine aminotransferase
AST: Aspartate aminotransferase
BMI: Body mass index
CRP: C-reactive protein
CV: Coefficient of variation
ECM: Extracellular matrix
ELISA: Enzyme-linked immunosorbent assay
FAK: Focal adhesion kinase
HDL-C: High-density lipoprotein cholesterol
HOMA-IR: Homeostatic Model of Assessment-Insulin Resistance
LDL-C: Low-density lipoprotein cholesterol
NAFLD: Non-alcoholic fatty liver disease
TG: Triglyceride
VLDL-C: Very low-density lipoprotein cholesterol

## References

[1] Farrell GC, Larter CZ (2006). Nonalcoholic fatty liver disease: from steatosis to cirrhosis. Hepatology.

[2] Jeanne MC, Frederick LB, Anna MD (2002). Nonalcoholic fatty liver disease. Gastroenterology.

[3] Mak KM, Mei R (2017). Basement membrane type iv collagen and laminin: an overview of their biology and value as fibrosis biomarkers of liver. Anat rec (Hokoben).

[4] Aumailley M, Smyth N (1998). The role of laminins in basement membrane function. J Anat.

[5] Gressner AM, Bachem MG (1990). Cellular sources of noncollagenous matrix proteins: role of fat-storing cells in fibrogenesis. Semin Liver Dis.

[6] Martinez-Hernandez A (1984). The hepatic extracellular matrix. I. Electron immunohistochemical studies in normal rat liver. Lab Invest.

[7] Hirayama C, Suzuki H, Takada A, Fujisawa K, Tanikawa K (1996). Serum type IV collagen in various liver diseases in comparison with serum 7S collagen, laminin, and type III procollagen peptide. J Gastroenterol.

[8] Younesi S, Parsian H (2019). Diagnostic accuracy of glycoproteins in the assessment of liver fibrosis: a comparison between laminin, fibronectin, and hyaluronic acid. Turk J Gastroenterol.

[9] Abeer MH, Yasser SS, Mohamed H, Shereen AE (2013). Could serum laminin replace liver biopsy as gold standard for predicting significant fibrosis in patients with chronic hepatitis b? Clinical and histopathological study. J Asian Sci Res.

[10] Dos Santos VN, Leite-Mór MMB, Kondo M, Martins JR, Nader H, Lanzoni VP (2005). Serum laminin, type IV collagen and hyaluronan as fibrosis markers in non-alcoholic fatty liver disease. Braz J Med Biol Res.

[11] Williams AS, David LK, Wasserman DH (2015). The extracellular matrix and insulin resistance trends. Endocrinol Metab.

[12] Geloneze B, Repetto EM, Geloneze SR, Tambascia MA, Ermetice MN (2006). The threshold value for insulin resistance (HOMA-IR) in an admixture population IR in the Brazilian metabolic syndrome study. Diabetes Res Clin Pract.

[13] McPherson S, Stewart SF, Henderson E (2010). Simple non-invasive fibrosis scoring systems can reliably exclude advanced fibrosis in patients with non-alcoholic fatty liver disease. Gut.

[14] Williams AS, David LK, Wasserman DH (2015). The extracellular matrix and insulin resistance. Trends Endocrinol Metab.

[15] Parnaud G, Hammar E, Rouiller DG, Armanet M, Halban PA (2006). Blockade of β1 integrin–laminin-5 interaction affects spreading and insulin secretion of rat β-cells attached on extracellular matrix. Diabetes.

[16] Bisht B, Goel HL, Dey CS (2007). Focal adhesion kinase regulates insulin resistance in skeletal muscle. Diabetologia.

[17] Bisht B, Srinivasan K, Dey CS (2008). In vivo inhibition of focal adhesion kinase causes insulin resistance. J Physiol.

[18] Hammar E, Parnaud G, Domenico B, Nadja P, Kathrin M (2004). Extracellular matrix protects pancreatic β-cells against apoptosis role of short- and long-term signaling pathways. Diabetes.

